# *Ehrlichia canis:* Is It a Pathogen for Humans and Other Primates?

**DOI:** 10.3390/pathogens15020236

**Published:** 2026-02-20

**Authors:** Valentina Virginia Ebani

**Affiliations:** 1 Department of Veterinary Sciences, University of Pisa, Viale delle Piagge 2, 56124 Pisa, Italy; valentina.virginia.ebani@unipi.it; 2 Centre for Climate Change Impact, University of Pisa, Via del Borghetto 80, 56124 Pisa, Italy

**Keywords:** *Ehrlichia canis*, human monocytic ehrlichiosis, non-human primates, zoonosis

## Abstract

Ehrlichioses and anaplasmosis are among the most commonly reported tick-borne diseases in humans and some animal species. *Ehrlichia canis* is the causative agent of canine monocytic ehrlichiosis; it primarily affects dogs and is usually transmitted by Rhipicephalus sanguineus ticks. Some reports suggest that this bacterium is a zoonotic pathogen capable of causing clinical symptoms consistent with human monocytotropic ehrlichiosis caused by *Ehrlichia chaffeensis*. Non-human primates seem to be susceptible to *E. canis*-infection, although it is not clear whether the bacterium can cause disease in these mammals. The number of cases of *E. canis* infections in human beings and other primates could be underestimated, mainly because inappropriate laboratory diagnoses are often carried out. Serological tests do not distinguish infection by *E. canis* from those due to other ehrlichial species; therefore, a correct diagnosis is possible only through molecular methods. Furthermore, *E. canis* is not usually recognized by veterinarians and clinicians as a possible pathogen of primates, and it is not included in the panel of tick-borne pathogens routinely investigated. Further studies are pivotal to verify the pathogenicity of *E. canis* in primates, and epidemiological investigations are needed to monitor its spread in animal and tick species not usually associated with this agent.

## 1. Introduction

Arthropod-borne diseases are infectious diseases frequently reported in humans and animals worldwide. They are caused by bacteria, viruses, and protozoa transmitted through the bites of hematophagous arthropods. Climate change in recent decades, mainly the increase in global temperature, has favored the expanding distribution of hematophagous arthropods across large areas of several continents, resulting in increased circulation of microorganisms and the presence of new pathogens in new geographic areas [1]. The spreading of ticks is markedly influenced by the new climatic conditions, and tick-borne pathogens are increasingly recognized as responsible for diseases in animals and human beings. Ehrlichioses are tick-borne diseases (TBDs) caused by members of the genus *Ehrlichia*, order *Rickettsiales*, which affect several mammal species. Among them, *Ehrlichia canis* is known as the primary causative agent of canine monocytic ehrlichiosis (CME), a severe and potentially fatal immunosuppressive disease transmitted globally by the brown dog tick, *Rhipicephalus sanguineus* sensu lato [2]. *Ehrlichia canis* has a worldwide distribution and is endemic in temperate and tropical regions of Europe, Africa, and the USA. A zoonotic role of *E. canis* has been supposed since the 1980s. In fact, although some cases initially attributed to *E. canis* were later proven to be caused by other ehrlichial species, *E. canis* has been detected in human patients with clinical signs resembling those of CME [3].

The aim of the present narrative review was to focus on cases of human *E. canis* infection reported in the literature in order to better understand the role of this agent as a human pathogen and to clarify the distinction between human infections truly caused by *E. canis* and those related to other monocytic ehrlichiae. In addition, attention was given to the potential role of *E. canis* in causing infection and disease in non-human primates.

## 2. Etiology

Ehrlichiae are small Gram-negative obligately intracellular bacteria that reside as microcolonies in vacuoles, particularly within monocytes/macrophages or polymorphonuclear leukocytes. Ehrlichiae are cocci that stain dark blue to purple with Romanovsky stain or light red with the Machiavello method. The replicative cycle of these bacteria is characterized by three development stages. The first stage is represented by small elementary bodies of 0.2–0.4 µm in diameter, the second by slightly larger initial bodies of 0.5–4 µm, and the third by even larger (4–6 µm) inclusion bodies. At the end of the cycle, a variable number of elementary bodies are grouped in a morula within a vacuole of host origin that separates the ehrlichiae from the host cell cytoplasm. The multiplication of the bacteria in the vacuole takes place via binary fission [4].

The genus *Ehrlichia* is included in the family Anaplasmataceae, order *Rickettsiales.* The family also encompasses the genera *Anaplasma*, *Neorickettsia*, *Neoehrlichia*, and *Wolbachia*. Members of the genus *Ehrlichia* are *E. canis*, *E. chaffeensis*, *E. ewingii.*, *E. japonica*, *E. minasensis*, *E. muris*, and *E. ruminantium*. Currently, several *Candidatus* Ehrlichia spp. are recognized as well [5]. Other species previously included in the genus *Ehrlichia* have been transferred into other genera, such as *E. sennetsu* and *E. risticii*, which now are classified as *Neorickettsia sennetsu* and *Neorickettsia risticii*, respectively, and *E. equi*, which is now renamed *Anaplasma phagocytophilum* [5].

*Ehrlichia canis* infection was first recognized in 1935 by Donatien and Lestoquard in military dogs, with symptoms of fever and anemia, in Algeria [6], but the first in vitro cultivation of the agent was carried out by Nyindo et al. in 1968 [7]. Between 1968 and 1970, a severe epidemic of canine ehrlichiosis, a term synonymous with tropical canine pancytopenia, resulted in the loss of hundreds of military working dogs in Vietnam [8,9]. Since then, *E. canis* has become largely recognized worldwide.

*Ehrlichia chaffeensis* is the causative agent of human monocytotropic ehrlichiosis. The disease was first described in 1986, but the etiologic agent was isolated in 1991 from a young man in Arkansas [10]. *Its main vector* is the lone star tick (*Amblyomma americanum*), and the main reservoir is the white-tailed deer (*Odocoileus virginianus*). Natural infection has also been reported in other mammals (goats, dogs, red foxes, coyotes) [11], while *E. chaffeensis* DNA has been detected in other tick species [12]. Currently, *E. chaffeensis* cases are only reported in the United States, where the vector is present [11].

*Ehrlichia ewingii*, once thought to be an exclusive canine pathogen, is now considered the etiologic agent of human granulocytic ehrlichiosis. It was first discovered by Ewing in 1970 [13]; the organism was observed in neutrophils from a febrile dog with mild clinical illness and was initially considered a novel strain of *E. canis* [13]. It was recognized as a human pathogen when it was related to illness in four patients initially suspected of having *E. chaffeensis* infection [14]. It is largely spread in USA, but it has also been detected in Africa and, recently, Europe (Hungary) [15].

*Ehrlichia minasensis*, a species closely related to *E. canis*, was initially reported in Brazil and Canada. This bacterium has now been reported in Pakistan, Malaysia, China, Ethiopia, and South Africa; in Europe, its presence has been demonstrated in Corsica [16]. It affects cattle, cervids, and dogs. Several tick species can act as vectors of this bacterium; in fact, it has been identified in *Rhipicephalus* spp., mainly *R. microplus*; *Amblyomma* spp. [17]; *Hyalomma* spp. [18,19]; and *Haemaphysalis* spp. [20].

*Ehrlichia japonica* was initially found in Japan and firstly identified as *Ehrlichia* sp. HF [21,22]. Successively, *E. japonica* was isolated from ticks of various *Ixodes* species in Japan, France, Serbia, and Romania. This species is highly pathogenic to mice [22].

*Ehrlichia ruminantium* (previously defined as *Cowdria ruminantium*) is the causative agent of heartwater disease in wild and domestic ruminants (cattle, sheep, goats); it is largely present in Africa and is transmitted by ticks of the genus *Amblyomma* [23].

*Ehrlichia muris*, isolated for the first time from a wild mouse in Japan [24], currently includes *E. muris* subsp. *eauclairensis* and *E. muris* subsp. *muris*. The agents predominantly affect rodents, but they were also reported as human pathogens [25].

## 3. Transmission

Dogs are known to be the main reservoirs of *E. canis*, but other wild canids such as foxes, wolves, and jackals can become infected [26]. Moreover, several studies have reported the presence of *E. canis* DNA in cats and wild felids [27].

Members of the genus *Ehrlichia* are transmitted through the bites of infected ticks belonging to different species and genera. The transmission of *E. canis* is usually due to the brown dog tick *R. sanguineus* s.l. This tick has been shown experimentally to be a competent vector for *E. canis*, which is transmitted trans-stadially, but not transovarially [28].

The transmission of *E. canis* by *R. sanguineus* ticks starts within 3 h after the tick’s attachment to the dog [29]. The development of *E. canis* in hemocytes, midgut epithelium, and salivary glands of engorging, adult *R. sanguineus* was previously demonstrated [30]. Transmission by adult *R. sanguineus* occurred only when the ticks engorged in larval or nymphal stages on dogs during the acute phase of infection. Attempts to transmit *E. canis* with adult ticks that fed to repletion as nymphs on dogs during the subclinical and chronic phases of infection were unsuccessful [31].

An experimental study highlighted the potential role of another tick species, *Dermacentor variabilis*, as a vector for *E. canis;* successful trans-stadial transmission was also demonstrated [32]. Recently, Rodriguez-Rojas et al. [33] found *E. canis* in 2/10 *D. variabilis* ticks in Mexico, confirming that this tick species can be involved, although marginally, in the epidemiology of *E. canis*.

Recently, *E. canis* DNA was detected in an engorged female *Haemaphysalis punctata* in southern Italy [34], but the real role of this tick species in the epidemiology of *E. canis* has not been elucidated. However, this finding suggests a need for monitoring other tick species, in addition to *R. sanguineus*, to assess the presence and spread of *E. canis* in a given environment.

The transmission of *E. canis* from one infected dog to another can occur through transfusions of infected blood from donors who have not been properly screened for this pathogen [35]. In addition, the transplacental vertical transmission of *E. canis* from naturally infected dams to their offspring has been proven to be possible, although uncommon [36]. This finding opens a new epidemiological view; in fact, the pathogen could persist in a canine population until the tick vector is reintroduced.

## 4. Monocytic Ehrlichiosis

### 4.1. Canine Monocytic Ehrlichiosis

CME can manifest in dogs in different clinical forms, ranging from mild to severe signs in relation to the phase of infection. The course of *E. canis* infection can be divided into acute, subclinical, and chronic phases, although these steps are not always clearly distinct in dogs with naturally occurring disease [37]. The acute phase, of about 2–4 weeks duration, is characterized by fever, anorexia, depression or lethargy, generalized lymphadenomegaly, splenomegaly, bleeding tendency, mucosal pallor, and ocular abnormalities, mainly uveitis. Additional clinical presentations in dogs during the chronic phase included ulcerative stomatitis with, in some cases, necrotic glossitis; hind limb and/or scrotal edema; and central nervous system signs such as seizures, ataxia, vestibular dysfunction, and cervical pain. Bleeding tendency continues during the chronic phases and is manifested as cutaneous and mucosal petechiae and ecchymoses, epistaxis, melena, hematuria, and prolonged bleeding from venipuncture sites; mortality due to septicemia is also observed. Clinical manifestations and/or hematological abnormalities may be absent or mild during the subclinical phase; splenomegaly, intermittent fever, thrombocytopenia, anemia may be observed in this phase, which may last months or years [37,38]. Occasionally, myelosuppression may develop without any premonitory signs indicative of the acute and subclinical phases of CME [39]. Therefore, the terms “myelosuppressive” and “non-myelosuppressive” CME are used to better indicate the disease severity. Spontaneous clinical recovery of acutely infected dogs may occur, but medical treatment is strongly recommended to prevent clinical exacerbation or death. It is unclear why some immunocompetent infected dogs eliminate the infection or remain sub-clinically infected with a low ehrlichiaemia level. Similarly, it has not been defined why some dogs develop the chronic illness phase [40].

### 4.2. Human Monocytic Ehrlichiosis

The name Human monocytic ehrlichiosis (HME) indicates disease due to monocytotropic ehrlichiae, in particular *E. chaffeensis*. HME usually starts as a febrile illness manifesting one to two weeks after the bite of an infected tick. The fever is accompanied by unspecific symptoms such as malaise, weakness, headache, myalgia, arthralgia, gastro-intestinal disorders with nausea, vomiting, diarrhea, and abdominal pain; sometimes, a rash is present. Hepatitis with an abnormal increase in hepatic transaminases can occur. In most cases, leukopenia and thrombocytopenia are observed [41].

In some severe cases, mainly in the absence of appropriate treatment, symptoms may include dyspnea, coagulopathy, and/or neurologic abnormalities due to the involvement of the central nervous system. These patients develop meningitis and/or have abnormalities in the cerebrospinal fluid. Consequent neurologic symptoms include confusion, photophobia, hyper-reflexia, ataxia, stiffness of the neck, seizures, and coma [41].

Even though many cases are asymptomatic or uncomplicated and self-limiting, severe complications, including septic shock, acute respiratory failure, meningoencephalitis, renal failure, and multi-organ failure, may occur and even be fatal. Increased severity and mortality are observed in immunocompromised patients, including the elderly [42].

## 5. *Ehrlichia canis* in Humans

A case of human infection attributed to *E. canis* was reported by Maeda and collaborators in 1987 [43]. They described a case of illness in a 51-year-old man admitted at a hospital with fever and confusion after being bitten by an unidentified tick in Arkansas. An acute clinical form started five days earlier with fever, malaise, headache, and myalgia; anemia and thrombocytopenia were also present. Blue-staining inclusions were observed in leucocytes of peripheral blood smears. A case of Rocky Mountain Spotted Fever (RMSF) caused by *Rickettsia rickettsii* was initially suspected; serum samples collected during the acute and convalescent phases were negative for antibodies to *R. rickettsii*, while a 1:640 antibody titer to *E. canis* antigen was detected via indirect immunofluorescence assay (IFA). In addition, occasional cytoplasmic inclusion bodies were observed in lymphocytes and monocytes in a peripheral blood sample [43].

During the same period, serological reactions to *E. canis* were found in some patients with febrile illness following known tick attachments in Georgia, Missouri, and South Carolina (USA), but the etiological agent was not isolated and/or identified [44]. Iin the following years, more accurate investigations revealed *E. chaffeensis* to be the etiologic agent involved in these cases, as well as in the case observed by Maeda et al. The initial misattribution of these cases to *E. canis* was due to inappropriate diagnostic procedures; in fact, IFA, with *E. canis* antigen, was employed, but it cannot distinguish common antigens shared by *E. canis* and *E. chaffeensis* [45]. Therefore, the identification of the ehrlichial species really involved in infection cases has only been possible since molecular analyses began to be applied.

Anderson and collaborators (1991) [10] proposed the name *Ehrlichia chaffeensis* sp. nov., with the Arkansas strain as the type strain, when they sequenced the *16S rRNA* gene amplified from the blood of two patients with human ehrlichiosis and from an isolate recovered from one of the patients. The comparison of these sequences with those of 16S rRNA of *E. canis* (two strains), *E. equi*/*E. phagocytophila* (two strains), *E. sennetsu* (two strains), and *E. risticii* indicated that the human ehrlichiosis agent was a new species most closely related to *E. canis* (homology of 98.2%) and more distantly related to other *Ehrlichia* spp. species [10].

In 1996, human infection by *E. canis* was first reported and demonstrated. The agent was isolated and molecularly characterized from an asymptomatic female patient in Venezuela [46]. A blood sample was collected from the patient, and morulae resembling those of ehrlichial organisms were seen in the cytoplasm of a few blood leukocytes. Subsequently, the leukocytes were overlaid on a monolayer of DH82 cells, and intracytoplasmic morulae were seen in the infected cells after 4 days. Electron microscopy showed that this ehrlichial isolate, called VHE (Venezuelan human ehrlichia), had an ultrastructural morphology compatible with those of *E. canis*, *E. chaffeensis*, and *E. muris*, but when the *16S rRNA* gene was amplified via polymerase chain reaction (PCR) and sequenced, the isolate was found to be only one to two bases different from *E. canis* strains Florida and Oklahoma, respectively (99.9% level of sequence similarity) [46].

Successively, *E. canis* was found in patients showing clinical signs compatible with ehrlichiosis in the same country [3]. In fact, based on the findings collected in 1996, twenty human patients with clinical signs compatible with human monocytic ehrlichiosis were hospitalized and carefully studied. Six patients were positive for *E. canis* 16S rRNA on gene-specific PCR, and sequencing analysis identified the etiological agent as identical to the VHE strain [3].

Moreover, Unver et al. (2001) [47] found 16S rRNA gene sequences identical to the VHE strain in dogs and ticks (*R. sanguineus*) in Venezuela; in addition, similar antigenic profiles between the dog and human isolates were observed. The findings suggested to the researchers that dogs can serve as a reservoir of human VHE infection and that *R. sanguineus* may serve as a vector.

A serological and molecular survey carried out in human blood donors in Costa Rica confirmed that *E. canis* may infect humans, even though it does not always cause clinical forms. The study detected 35% (35/100) of seropositive reactions with IFA, in some cases with high antibody titers ranging from 1:1024 to 1:8192. Moreover, 3.6% (10/280) of the blood specimens tested via PCR had *E. canis* DNA. In this study, analyses of the ehrlichial *trp36* polymorphic gene were carried out in the positive samples, and different *E. canis* genotypes, including a novel genotype, were identified [48].

In August 2023, a case of *E. canis* infection was confirmed in a 42-year-old male patient in Italy with a tick attached to his neck. The man had noticed the attached tick 48 h after a hike in a rural area of Campania region in southern Italy. The tick was identified as an engorged female belonging to the species *Haemaphysalis punctata*, as confirmed via molecular analyses. PCR detected a fragment of the *E. canis 16S rRNA* gene and a fragment of the *groEL* gene in DNA extracted from both the tick and the patient’s blood. Analyses of the two 16S rRNA amplicons found that the sequences were identical to each other and to those of *E. canis* obtained from a red fox (*Vulpes vulpes*) in the same study area, humans from USA and Venezuela, and dogs from Mediterranean Basin countries, such as Italy, Greece, Israel, Egypt, and Turkey [34]. Some days after the tick attack, the patient developed mild symptoms such as fever, headache, malaise, and muscle pain, which disappeared within a week without antimicrobial treatment [34].

Table 1 summarizes the cases of human ehrlichiosis attributed to *E. canis*. Despite these findings, currently, *E. canis* is not considered to be a typical zoonotic agent; thus, it is not usually investigated in humans, and the number of infection cases could be underestimated.

## 6. *Ehrlichia canis* in Non-Human Primates

The ability of *E. canis* to cause infection and disease has also been investigated in non-human primates. The first study was carried out by Van Harden and Goosen in 1981 [49], who injected blood collected from an *E. canis*-infected domestic dog into three adult vervet monkeys (*Cercopithecus pygerythrus)*. The monkeys did not seem to be susceptible to the pathogen. They did not develop any clinical or hematological signs of disease; in addition, they did not seem to be able to harbor the ehrlichiae, because when their blood was collected 28 days post-infection and injected intravenously into two dogs, these animals showed no signs of disease and peripheral blood smears failed to demonstrate morulae of *E. canis.* Furthermore, neither morulae of *E. canis* nor hematological evidence of ehrlichiosis were seen after these monkeys had been splenectomized [49].

In the period 2010–2012, a study was conducted in free-ranging marmosets, genus *Callithrix*, living in an Atlantic Forest fragment in the municipality of Viçosa, state of Minas Gerais, Brazil. Blood samples were collected from 19 marmosets and analyzed via PCR for *E. canis.* One of the samples analyzed was positive; the amplified product was sequenced and subjected to BLAST analysis, showing 100% similarity with several *E. canis* sequences stored in GenBank. In particular, the strain found in the marmoset was homologous to others previously identified in several hosts, such as domestic dogs (*Canis lupus familiaris*), wild cats (*Prionailurus bengalensis euptilurus*), Iriomote cats (*Prionailurus iriomotensis*), small spotted cats (*Leopardus tigrinus*), ocelots (*Leopardus pardalis*), and ticks (*R. sanguineus*) in various countries, including Brazil, Tunisia, Thailand, Cape Verde, Taiwan, Italy, Malaysia, Philippines, India, and Romania [50]. The marmoset that tested positive did not show clinical signs; the detection of *E. canis* DNA in this animal is not enough to assess the pathogenic role of the agent and to determine whether the primate is an accidental host or can act as a reservoir. The tested marmosets had no ectoparasites when they were captured for sampling [50]; therefore, it could not be determined if *R. sanguineus* or other hematophagous arthropods were responsible for transmission.

Recently, *E. canis* was found in a primate in Brazil. In detail, 38 samples of six non-human primate species (*Alouatta caraya, Aotus azarae, Aotus infulatus, Ateles marginatus, Mico melanurus*, and *Sapajus apella)* were collected in Mato Grosso State, Brazil, and analyzed through PCR for ehrlichial agents. One free-living tufted capuchin (*S. apella*) tested positive for *Ehrlichia* spp., and sequencing analyses of different genes (*dsb*, *groEL*, *16S rRNA*, and *gltA*) identified 100% homology with multiple sequences of *E. canis* detected in domestic dogs from Colombia, Myanmar, Argentina, and Brazil. Furthermore, DNA of an ehrlichial strain showing 100% homology with *E. chaffeensis* from the USA and Mexico was detected in one black-tailed marmoset *(M. melanurus)* [51]. In both cases, it was not possible to assess the role of the infected animals in the epidemiology of *E. canis* and *E. chaffeensis* and whether these bacterial agents can cause disease.

Other *Ehrlichia* species were found in non-human primates as well. A naturally occurring infection caused by *E. chaffeensis* in ring-tailed lemurs (*Lemur catta*) and ruffed lemurs (*Varecia variegata*) in North Caroline (USA) was described. The DNA of the pathogen was identified via PCR in peripheral blood from six of eight clinically ill lemurs. Organisms were also cultured from the blood of one lemur exhibiting clinical and hematologic abnormalities like those of humans infected with *E. chaffeensis* [52].

The occurrence of *Ehrlichia* spp. infection in an anemic langur (*Semnopithecus* sp.) from Nagpur District of Maharashtra State, India, was reported in 2009. Blood smears were prepared from the heart blood of the animal dead for trauma and stained with Leishman’s stain; the microscopic examination revealed *Ehrlichia* spp. organisms in monocytes, but the pathogen species was not identified [53].

In another investigation, PCR assays for detecting *A. phagocytophilum*, *E. chaffeensis*, *E. ewingii*, and *E. canis* were conducted on blood samples from 56 lemurs (*L. catta*, *Eulemur macaco flavifrons*, *V. variegata variegata*) living in St. Catherines Island, Georgia. Only two *L. catta* were positive for *E. chaffeensis*, but no animal developed clinical signs, except for slight hypoproteinemia and moderately high serum alanine aminotransferase levels in one lemur [54]. The finding of antibodies to *A. phagocytophilum* and *E. chaffeensis* in 38.5% (20/52) and 30.8% (16/52) of the adult lemurs analyzed, respectively, confirms the exposure of these animals to the investigated arthropod-borne bacteria. The presence of ticks harboring ehrlichial agents were largely demonstrated in this geographic area [55]; therefore, it is supposable that lemurs became infected after tick bites, although it is not clear what tick species was involved. The role of ticks in the transmission of ehrlichiae to primates was also demonstrated by Lacroux et al. [56]. The researchers assessed the presence of ticks and tick-borne pathogens in the habitat of wild chimpanzees in western Uganda, where domestic animals are banned. A total of 470 ticks were collected and identified as *Haemaphysalis parmata* (68.77%), *Amblyomma tholloni* (20.70%), *Ixodes rasus* sensu lato (7.37%), *Rhipicephalus dux* (1.40%), *Haemaphysalis punctaleachi* (0.70%), *Ixodes muniensis* (0.70%), and *Amblyomma paulopunctatum* (0.35%). Molecular analyses revealed the presence of at least six genera of tick-borne pathogens (*Babesia*, *Theileria*, *Borrelia*, *Cryptoplasma*, *Ehrlichia*, and *Rickettsia*). *Ehrlichia* spp. DNA was found in one *H. punctaleachi*; the sequencing analysis revealed that this agent was closely related to *Candidatus* Ehrlichia shimanensis, which is closely related to *E. canis* [56]. The cases of monocytotropic ehrlichiosis diagnosed in non-human primates are reported in Table 2.

## 7. Discussion

Data about ehrlichiosis in non-human primates, particularly the form caused by *E. canis*, are very scant. Overall, tick-borne infections are not usually investigated in these mammals because of the difficulty in collecting samples (blood and/or ticks).

Wild non-human primates, such as marmosets in Brazil, which often live in areas adjacent to urbanized habitats, and they have come into increasing contact with humans and domestic animals, sometimes creating conflict between human needs and wildlife habitats and health [50]. Therefore, investigations of tick-borne pathogens in wild non-human primates living in habitats close to humans could contribute to evaluating the spreading of these pathogens in a given geographic area. The sporadic detection of *E. canis* in these mammals has not clarified their role as accidental hosts, natural reservoirs, or even amplifiers of the pathogen.

Currently, the data available in the literature do not clarify whether these mammals develop disease. No clinical signs were observed in marmosets experimentally infected with *E. canis* and in other non-human primates in which ehrlichiae were detected. Several factors could influence the course of *Ehrlichia*-infection, such as primate species, the host’s immune system, the infecting dose, and the *E. canis* strain.

Non-human primates have protective methods to remove their ectoparasites, including ticks; in fact, they spend a considerable amount of time self-grooming and allo-grooming, which have important hygienic functions [57]. Consequently, it is conceivable that tick-borne infections are not frequent in these animals, but the risk of infection could increase in areas with high tick density.

Regarding the role of the brown dog tick *R. sanguineus*, considered the main vector of *E. canis*, it appears to sporadically parasitize non-human primates. Some reports have identified *R. sanguineus* on black howler monkey (*Alouatta caraya*), southern brown howler monkey (*Alouatta guariba clamitans*), red-faced spider monkey (*Ateles paniscus*), *Callitrix* sp., and marmoset in Brazil [58]. *Ehrlichia canis* could become a threat to all primates in environments regularly frequented by dogs and other canids, which often host infected ticks.

Ehrlichioses, together with anaplasmosis, are among the tick-borne diseases most frequently reported in humans, mainly on certain continents, after Lyme disease [42,59,60]. *Ehrlichia chaffeensis*, the causative agent of HME, is the *Ehrlichia* species most frequently observed in humans on the American continent, but other species, such as *E. ewingii* and *E. muris* subsp. *eauclairensis*, can also cause diseases and are often detected in North America. Even though these *Ehrlichia* species are considered human pathogens, they can also infect different animal species that usually act as reservoirs. In all cases, pathogens are transmitted to humans, as well as animals, by tick bites.

Other ehrlichial species are sporadically detected in human samples and are considered responsible for clinical forms in patients. Three fatal cases, one woman and two children, were reported in South Africa and were associated with *E. ruminantium* [61]. Similarly, *Candidatus* Ehrlichia cajennense has been suspected to pose a potential threat to humans in South America, because it was found in the Cayenne tick (*Amblyomma cajennense*), which is widely distributed in this geographic area, and virulence factors similar to those of *E. chaffeensis* were identified [62].

In Europe, granulocytic ehrlichiosis related to *A. phagocytophilum* is considered one of the most frequently encountered human tick-borne diseases, whereas the monocytic form is considered absent; in fact, *E. chaffeensis* has never been found in patients, animals, or ticks in European countries. Nevertheless, a case of *E. canis* infection has been recently reported in Italy in a man with disease [34]. This single case is not sufficient to determine the pathogenic role of *E. canis* for humans, just as its detection in *H. punctata* cannot confirm the epidemiological role of this tick species. However, the Italian case highlights the changing epidemiology of TBDs, with the occurrence of new pathogens in a given area or infection caused by known agents in new hosts, due to the expanding geographic range of the ticks and the reservoirs, mainly driven by climatic changes. From this perspective, it is essential to stay updated on the epidemiological evolution of tick-borne pathogens, and clinicians should consider these infections, including those by *Ehrlichia* spp., when managing patients with influenza-like illnesses, primarily in endemic areas.

A prompt and correct diagnosis of ehrlichiosis is the main step to resolve the disease and to avoid complications, mainly in elderly and immunocompromised patients.

Diagnosis of ehrlichiosis requires a comprehensive approach, starting with accurate anamnesis, which must include details about trips to endemic areas, outdoor activities, and occupational environments that may increase exposure to tick bites. A correct diagnosis requires laboratory techniques, which include serologic analysis (IgM and IgG antibodies detected via indirect immunofluorescence assay), PCR, immunohistochemistry, and the identification of morulae in monocytes (for human monocytic ehrlichiosis) or neutrophil granulocytes (for human granulocytic ehrlichiosis) in peripheral blood or buffy coat smears [63]. IFA is the most commonly employed serological test able to detect IgM and IgG antibodies. It has good sensitivity, but it cannot distinguish antibodies against the different monocytotropic *Ehrlichia* species that share common antigens [45]. In fact, the presence of a small subset of outer membrane proteins is common in *E. canis* and *E. chaffeensis*; these antigens react strongly with antibodies in sera from infected patients, and it is possible to distinguish them only through more specific tests such as Western immunoblotting or Enzyme-Linked Immunosorbent Assay (ELISA) [64]. Immunohistochemistry is possible when tissue samples are available; observation of blood/buffy coat smears is not very sensitive and does not recognize the *Ehrlichia* species. PCR is highly sensitive, and it allows for identifying the ehrlichial species; therefore, it is considered the standard method for diagnosis [63]. Usually, a single genetic target of *E. canis* or *E. chaffeensis* (*16SrRNA*) is searched for via PCR, but protocols for the detection of genes, such as *dsb*, *groEL*, *gltA*, and *trp36*, can increase the specificity of the diagnosis and are used for phylogenetic analyses [51].

## 8. Conclusions

The sporadic cases of infection by *E. canis* observed in humans and other primates suggest that this tick-borne pathogen, traditionally associated with dogs, could also affect these mammals. Information reported in the literature has not established zoonotic transmission cycles. Moreover, further studies are needed to better assess the ability of *E. canis* to cause disease in primates. Studies addressing these gaps could provide the information necessary to promptly propose and diagnose this infection. In addition, epidemiological studies are pivotal for the continuous monitoring of the spread of *E. canis* in a given area, as well as in animal and tick species, including those not typically associated with this bacterium.

## Figures and Tables

**Table 1:** Monocytotropic ehrlichiosis cases in humans attributed to *Ehrlichia canis*.

Case	Country	Clinical Signs	Diagnostic Analyses	Species Really Involved	Ref.
Man	Arkansas (USA)	yes	IFA (*E. canis* antigen)	*E. chaffeensis*	[43]
Unspecified individuals	Georgia, Missouri (USA), Sud Carolina (USA)	yes	IFA (*E. canis* antigen)	*E. chaffeensis*	[44]
Woman	Venezuela	no	Isolation + molecular analysis	*E. canis*	[46]
Unspecified individuals	Venezuela	yes	PCR *	*E. canis*	[3]
Unspecified individuals	Costa Rica	no	IFA (*E. canis* antigen) PCR **	*E. canis*	[48]
Man	Italy	yes	PCR ***	*E. canis*	[34]

Legend. IFA: indirect immunofluorescence assay; PCR: polymerase chain reaction; *: *16SrRNA*; **: 16SrRNA and *trp36*; ***: *16SrRNA* and *groEL.*

**Table 2:** Monocytotropic *Ehrlichia*—infections diagnosticated in non-human primates.

Animal		Country	Clinical Signs	Diagnostic Analyses	Species Involved	Ref.
Lemur	*(Lemur catta)*	Georgia	Hypoproteinemia, high ALT	PCR	*E. chaffeensis*	[54]
Langur	*(Semnopithecus* sp.)	India	Anemia	Leishman’s stain	*Ehrlichia* spp.	[53]
Marmoset	*(Callithrix* sp.)	Brazil	no	PCR * + sequencing	*E. canis*	[50]
Capuchin	*(Sapajus apella)*	Brazil	no	PCR ** + sequencing	*E. canis*	[51]
Marmoset	*(Mico melanurus)*	Brazil	no	PCR ** + sequencing	*E. chaffeensis*	[51]

Legend. ALT: alanine aminotransferase; PCR: polymerase chain reaction; *: gene target 16SrRNA; **: *16S rRNA*, *dsb*, *groEL*, *gltA.*

## Data Availability

Data are reported in the manuscript.
